# Involvement of Angiopoietin-2 and Tie2 Receptor Phosphorylation in STEC-HUS Mediated by* Escherichia coli* O104:H4

**DOI:** 10.1155/2015/670248

**Published:** 2015-12-24

**Authors:** Alexander Lukasz, Jan Beneke, Kristina Thamm, Jan T. Kielstein, Jan Menne, Jan-Henrik Mikesch, Bernhard M. W. Schmidt, Hermann Haller, Philipp Kümpers, Sascha David, Mario Schiffer

**Affiliations:** ^1^Department of Nephrology & Hypertension, Hannover Medical School, Carl-Neuberg Strasse 1, 30625 Hannover, Germany; ^2^Department of Medicine, Division of General Internal Medicine, Nephrology, and Rheumatology, University Hospital Münster, Albert-Schweitzer-Strasse 33, 48149 Münster, Germany; ^3^Department of Hematology, Hemostaseology, Oncology and Pneumonology, University Hospital Münster, Albert-Schweitzer-Strasse 33, 48149 Münster, Germany

## Abstract

*Escherichia coli* O104:H4-associated hemolytic uremic syndrome (HUS) is characterized by Shiga toxin-induced vascular damage. As indicated by recent studies, dysregulation of the angiopoietin (Angpt)/Tie2 ligand receptor system may be crucial for endothelial dysfunction in HUS. Early Angpt-2 levels quantified in 48 adult HUS patients were predictive for a complicated clinical course, in particular for need of hemodialysis and mechanical ventilation as well as occurrence of seizures.* In vitro* challenge of human umbilical vein endothelial cells with patients' sera indicated an injurious mediator role of Angpt-2 opening future perspectives for mitigating endothelial activation in HUS.

## 1. Introduction

Hemolytic uremic syndrome (HUS) is characterized by acute hemolytic anemia, thrombotic microangiopathy, and kidney injury often caused by intestinal infections with Shiga-like toxin-producing* E. coli* (STEC-HUS). STEC-HUS is a medical emergency with a mortality rate of 4% [1]. A significant proportion of patients suffer from complications such as bloody diarrhea, neurological symptoms up to generalized seizures, and acute kidney injury (AKI) or need for renal replacement therapy (RRT) [1]. Many patients with STEC-HUS show spontaneous remission and complete recovery within a few weeks. However, in some patients the disease progresses rapidly requiring best supportive treatment, intensive care, and interventional therapy. Definition of relevant prognostic parameters for early identification of high-risk patients remains a challenge for treating physicians. Instruments of risk evaluation such as prognostic biomarkers remain highly desirable.

Global endothelial damage of the microvasculature and subsequent organ failure is pathophysiological hallmark of STEC-HUS [2]. Some pathological details remain unidentified, while Shiga toxins are known to alter expression of chemokines, chemokine receptors, and molecules of cell adhesion, thereby increasing leucocyte recruitment [3]. In addition, platelets [4] and neutrophil granulocytes [5] are activated. Endothelial reactions include an increased release of tissue-factor [6] and activation of several pathways [7]. Concerning endothelial activation, we also focused on a potential involvement of angiopoietins.

Angiopoietin-1 (Angpt-1) and Angpt-2 are antagonistic ligands of the endothelial tyrosine kinase Tie2. The Angpt/Tie2 ligand receptor system is a nonredundant gatekeeper of endothelial activation and controls the endothelial phenotype during angiogenesis and inflammation. Angpt-1 is continuously released by pericytes and maintains endothelial quiescence. In contrast, Angpt-2 competitively inhibits binding of Angpt-1 to Tie2 and thereby disrupts protective Angpt-1 signalling, causing loss of vessel integrity, vascular leakage, and expression of inflammatory genes [8]. In a recent study published, the dysregulation of the Angpt/Tie2 system was associated with a diagnosis of STEC-HUS in children [9]. However, clinical and prognostic impact of this phenomenon still remains elusive, and no data is available assessing the role of circulating angiopoietins in adult STEC-HUS patients.

On a single centre basis, we analyzed Angpt-2 plasma levels in adult STEC-HUS patients during the largest reported outbreak of* Escherichia coli* O104:H4 in northern Germany from May till July 2011 [10, 11]. We aimed to determine early predictive factors for complications and outcome.

## 2. Materials and Methods

### 2.1. Patients and Study Design

Forty-eight patients with STEC-HUS treated in Hannover Medical School during the outbreak in 2011 were included in this study after obtaining written informed consents from the patients or their legal representatives. The study was performed in accordance with the declaration of Helsinki and approved by the Institutional Review Board at Hannover Medical School (number 1123-2011).

STEC-HUS was diagnosed when patients had EHEC and/or Shiga toxin-2 positive stools or history of bloody diarrhoea between May and July 2011 and fulfilled all of the following three criteria: (1) platelet count below 150/nL, (2) haemolytic anaemia with haemoglobin below the lower limit of normal, and (3) kidney injury as indicated by an increase of urea or creatinine [12]. All of the above patients fulfilled these criteria.

### 2.2. Blood Sampling and Quantification of Circulating Angpt-1 and Angpt-2

EDTA plasma samples for quantification of Angpt-1 and Angpt-2 were obtained for each patient on different time points (admission and day three after admission or referral to our tertiary care hospital), immediately centrifuged, divided into aliquots, and stored at −80°C. Plasma Angpt-1 and Angpt-2 were measured as described previously [13]. Plasma samples of controls were also anticoagulated with EDTA.

### 2.3. Endothelial Cell Culture

Passage 5 human umbilical vein endothelial cells (HUVECs) (Lonza, Basel, Switzerland) were cultured, treated, and blotted as described previously in detail [14].

In order to perform fluorescent immunocytochemistry HUVECs were grown to confluence on glass coverslips, fixed, permeabilized, and stained with an anti-VE-cadherin antibody and with phalloidin to visualize F-actin as described previously [15]. These experiments were each time done in duplication and the results were reproducible (data not shown).

Endothelial cells were incubated with EBM-2 containing 5% fetal bovine serum (FBS) to grow to a confluent monolayer. When the experiment was started the tissue culture medium was changed to EBM-2 containing 5% of patients' plasma.

### 2.4. Measurements of Tie2

Tie2 and phosphoTie2 (pTie2) were measured as described previously [16, 17]. Shortly, antibodies against pTie2 (Y992) (R&D systems, Minneapolis, MN) and Tie2 (C-20) (Santa Cruz Biotechnology, Heidelberg, Germany) were used. For immunoblotting, equal amounts of lysed protein were resolved on 0% polyacrylamide gel and transferred to polyvinylidene fluoride (PVDF) membranes (Merck Millipore, Darmstadt, Germany). Membranes were blocked for 1 hour at room temperature in 3% bovine serum albumin (BSA). All washing steps were carried out in TBST (20 mM Tris, 150 mM NaCl, and 0.1% Tween 20 (Merck)). Afterwards, membranes were incubated with anti-phosphotyrosine antibody (R&D systems) or anti-Tie2 antibody (Santa Cruz). Proteins were visualized by secondary antibodies conjugated to horseradish peroxidase (HRP) (Santa Cruz) and by enhanced chemiluminescence. An exemplary blot from HUVEC lysates stimulated plasma from our patients is shown in Supplemental Figure  1 in Supplementary Material available online at http://dx.doi.org/10.1155/2015/670248.

### 2.5. Statistical Analysis

Continuous variables are expressed as medians with corresponding 25th and 75th percentiles (Interquartile Range (IQR)) and compared by using the Mann-Whitney rank sum test. Correlations between variables were assessed by Spearman's correlation test or linear regression. Data analysis and figure preparation were performed using GraphPad Prism (GraphPad Prism Software Inc., San Diego, California, USA).

## 3. Results

### 3.1. Patients' Characteristics

Our STEC-HUS cohort contained 48 patients (73% female) with a median age of 45 (27–57) years. None had a history of chronic kidney disease. All patients developed haemolysis, thrombocytopenia, and moderate to severe inflammation (Table 1). Median admission creatinine was 259.5 (163–342) *μ*mol/L and 43 patients (89.6%) suffered acute kidney injury (AKI) AKIN I or higher.

### 3.2. Angiopoietin Levels

Compared to healthy control patients, we found significantly lower plasma levels of Angpt-1 in STEC-HUS patients (1.36 [0.95–2.29] versus 2.5 [1.5–3.4] ng/mL, *p* < 0.05) as well as significantly elevated Angpt-2 (2.6 [1.8–3.28] ng/mL versus 1.39 [0.91–1.64], *p* < 0.0001, resp.) on day 3 after admission.

### 3.3. Association of Angiopoietins with a Complicated Clinical Course of STEC-HUS

**Figure 1:**
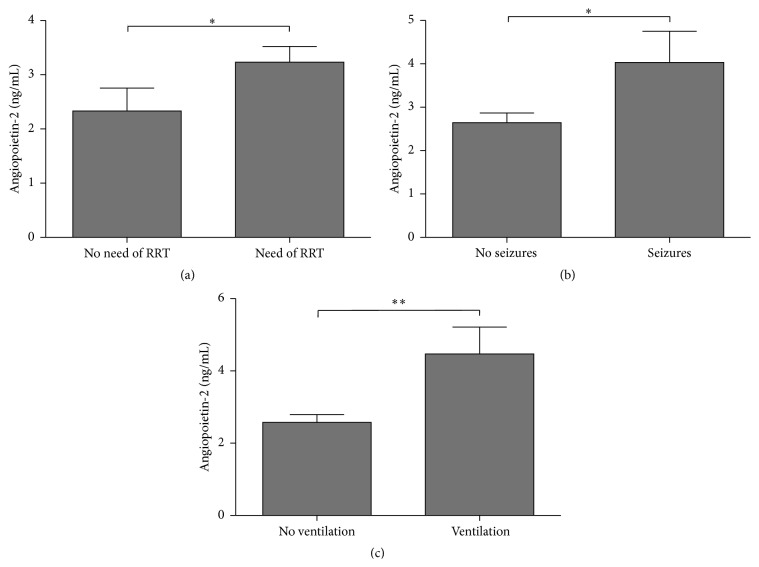
Association of Angpt-2 levels on day three after admission with clinical complications. Boxplots showing associations between Angpt-2 levels and clinical outcomes ((a) need of RRT, (b) occurrence of seizures, and (c) need of mechanical ventilation); ^*∗*^ *p* < 0.05 and ^*∗∗*^ *p* < 0.01.

Focusing on STEC-HUS patients, we compared Angpt-1 and Angpt-2 levels within subcollectives following different outcome parameters. In particular, early plasma levels of angiopoietins were then compared between patients being either more severely or less afflicted by clinical symptoms and complications during the course. 32 patients required RRT (66.7%) a median of 5 (3–7.3) days after admission. Angpt-2 levels were significantly higher in patients requiring RRT compared to those without (2.9 [2.1–3.8] ng/mL versus 1.8 [1.2–2.8] ng/mL, *p* < 0.05) (Figure 1(a)). Angpt-2 levels on day three after admission discriminated between patients requiring RRT and those who did not with an area under the receiver operating characteristic curve (ROC) of 0.73 (95% confidence interval, 0.567–0.888; *p* = 0.01) (data not shown). Even on day of admission, Angpt-2 levels were associated with patients requiring RRT compared to those without (2.7 [2.2–3.6] ng/mL versus 1.9 [1.3–2.2] ng/mL, *p* < 0.001) (data not shown).

A median of 10.5 days after admission, 10 patients (20.8%) developed neurological complications ranging from mild cognitive disorder to generalized seizures. Again, early Angpt-2 on day three was significantly increased in those patients with seizures during the course compared to those without (3.1 [2.5–5.7] ng/mL versus 2.4 [1.7–3.1] ng/mL, *p* < 0.05) (Figure 1(b)). The corresponding ROC curve showed an area under the curve (AUC) of 0.71 (95% CI, 0.547–0.882, *p* = 0.04) (data not shown).

Mechanical ventilation was needed in 9 patients (18.8%). Angpt-2 on day three after admission was higher in patients requiring mechanical ventilation compared to those without (3.8 [2.7–5.9] ng/mL versus 2.3 [1.7–3.1] ng/mL, *p* < 0.001) (Figure 1(c)). The AUC of the corresponding ROC curves was 0.80 (95% CI, 0.652–0.952, *p* = 0.005) (data not shown).

Angpt-2 levels on admission did not correlate with occurrence of seizures and need of mechanical ventilation (data not shown). Angpt-2 levels on admission did not correlate with occurrence of seizures and need of mechanical ventilation (data not shown). On admission and day three, neither Angpt-1 nor the Angpt-2/Angpt-1 ratio was associated with need of RRT, mechanical ventilation, or occurrence of seizures (data not shown).

### 3.4. Disruption of Endothelial Integrity* In Vitro* Might Be Induced by Angpt-2

**Figure 2:**
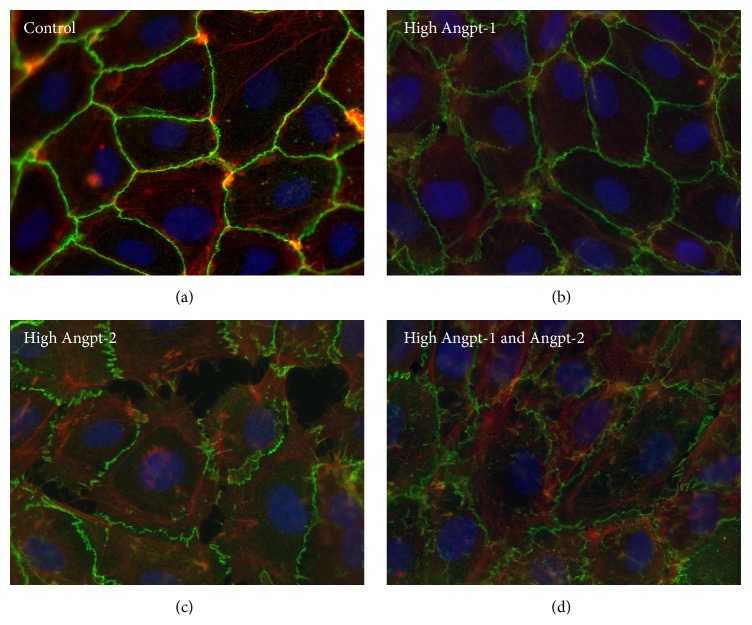
Fluorescent immunocytochemistry microscopy images showing immunofluorescence staining for F-actin (red) and VE-cadherin (green) performed on 100% confluent P5 HMVECs. Cells were treated with 5% prefiltered human EDTA plasma. EDTA plasma samples were collected from healthy controls (a), patients with high Angpt-1 (Angpt-1: 15.5 and 17.2 ng/mL) (b), high Angpt-2 (Angpt-2: 13.6 and 15.6 ng/mL) (c), and both high Angpt-1 and Angpt-2 (Angpt-1: 12.1 and 10.1 ng/mL, Angpt-2: 9.3 and 12.9 ng/mL, resp.) (d).

Parikh et al. have previously shown that, in sepsis, excess Angpt-2 diminishes Tie2 activation, which leads to paracellular gap formation driven by actin-myosin-based contraction of the cytoskeleton. Addition of Angpt-1 reverses this process [18]. To explore a potential mediator role of Angpt-1 and Angpt-2 we challenged endothelial cells with patients' plasma. We chose plasma from patients with high Angpt-2 levels (Angpt-2: 13.6 and 15.6 ng/mL) and challenged ECs to test the effect of higher Angpt-2 levels on the endothelial phenotype. Fluorescent immunocytochemistry for F-actin, a structural protein of the cytoskeleton, and VE-cadherin, a main constituent of endothelial adherens junctions, were performed. ECs treated with controls' plasma did not affect the confluent, adjacent cell monolayer with cortical actin architecture and the typical cell border localization of VE-cadherin. The same quiescent phenotype was observed using plasma from those HUS patients with a particularly high Angpt-1 concentration (Angpt-1: 15.5 and 17.2 ng/mL) (Figure 2). However, patients' plasma with a high Angpt-2 (Angpt-2: 13.6 and 15.6 ng/mL) disrupted the endothelial architecture, as depicted by increased actin stress fibers (ASF) and distinct endothelial gap formation. We then performed an experiment to test the effect of plasma with high Angpt-1 and Angpt-2 levels on the endothelial integrity. Exposure of patient's plasma with both high Angpt-1 and Angpt-2 levels (Angpt-1: 12.1 and 10.1 ng/mL, Angpt-2: 9.3 and 12.9 ng/mL, resp.) completely abolished the formation of ASF and interendothelial gaps.

### 3.5. Phosphorylation of the Tie2 Receptor* In Vitro*

**Figure 3:**
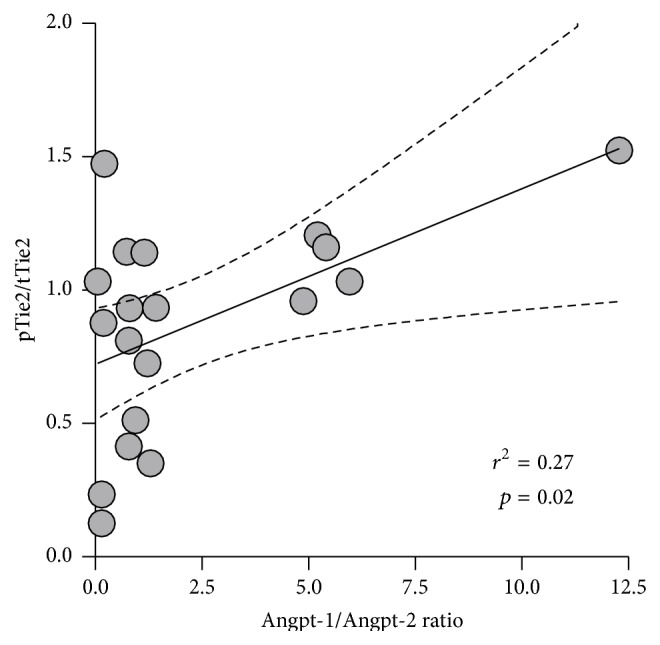
Linear regression shows the correlation between the Angpt-1/Angpt-2 ratio and the phosphorylated Tie2/total Tie2 ratio (pTie2/tTie2).

Ligation of Angpt-1 to Tie2 leads to its tyrosine phosphorylation, whereas Angpt-2 can antagonize Tie2 activation. To test whether or not gap formation is consistent with degree of Tie2 phosphorylation, we immunoblotted EC lysates for pTie2 after having been challenged with the Angpt-1 and Angpt-2 plasma constellations mentioned. The Angpt-1/Angpt-2 ratio was significantly associated with the phosphorylated Tie2/total Tie2 ratio (pTie2/tTie2) (*r* ^2^ = 0.27, *p* = 0.02) (Figure 3) indicating that these circulating molecules were indeed biologically active and that they target their canonical common receptor as a pathophysiological feature of HUS-associated endothelial dysfunction.

## 4. Discussion

Although STEC-HUS is categorized as a thrombotic microangiopathy, the incidences of thrombotic events or embolism were rare in the German outbreak 2011 [19]. Renal impairment seems to be also provoked by direct acute tubular damage even when thrombotic events are foreclosed by choice of mouse models [20]. In addition, the contribution of microvascular damage to clinical symptoms in STEC-HUS was indicated by prolonged T2 relaxation times in quantitative MRI scans of patients with neurological complications [21]. For the purpose of investigating mechanisms of endothelial damage and dysfunction in our patients, we correlated angiopoietin levels with outcome parameters.

From our data, we conclude that angiopoietins and Tie2 receptor alterations contribute to the early stage of STEC-HUS. It appears capable of indicating the severity of potential initial endothelial damage and thereby also a prognostic value for the onset of further complications. Affliction of endothelial integrity might be more important than thrombotic events, lightening a new aspect on this disease.

The endothelial integrity is supported by high Angpt-1 levels and impaired by elevated Angpt-2. Endothelial cells exposed to plasma with low Angpt-2 and high Angpt-1 levels showed intact cell-cell contacts and a cortical configuration of the cytoskeleton whereas exposure to high Angpt-2 without elevated Angpt-1 levels induced severe gap formation. In particular, the contribution of Angpt-2 is emphasized in our results, predicting the requirement of RRT and mechanical ventilation as well as occurrence of seizures at an early stage of the disease. As a Weibel-Palade body-stored protein, Angpt-2 can be rapidly released upon various stimuli including cytokines, thrombin, hypoxia, activated leucocytes, and platelets [22]. In children with* E. coli* O157:H7 mediated STEC-HUS early dysregulation of angiopoietins has been described recently [9]. During the pre-HUS phase, children exhibit prothrombotic coagulation abnormalities with evidence of early vascular injury preceding HUS with renal injury [23]. However, triggering factors inducing Angpt-2 release are still largely unknown, and clinical and prognostic impact of Angpt-2 plasma levels in STEC-HUS patients have been missing so far. A possible confounder of endothelial barrier disruption could be the presence of EDTA. By analogous confrontation of samples from patients and controls with EDTA, EDTA specific effects should be minimized from the observed differences.

Plasma levels of Angpt-1 showed no significant correlation with severity of affliction in our patients. Due to the sudden initiation of our study during the German outbreak 2011, Angpt-1 measurements could not be performed widely enough and thereby present a limitation to our study. As being expressed more constitutively by pericytes and vascular smooth muscle cells [22, 24], the adaption to a rapid increase of Angpt-2 is likely following delayed to clinical complications. Whether or not the administration of Angpt-1 to individuals afflicted by STEC-HUS could protect from endothelial, renal, or neurological impairment could be a valuable target of future studies. Other studies underlining diagnostic and prognostic potential of circulating Angpt-1 and Angpt-2 in various infectious diseases like malaria and/or sepsis with and without multiorgan dysfunction syndrome (MODS) [13, 18, 25, 26] indicate that detailed investigations of prognostic and therapeutic options for angiopoietins and the Tie2 receptor are worth considering intensely.

Our conclusions are that* E. coli* O104:H4 induced STEC-HUS is likely to be associated with early elevations of Angpt-2 and disruption of endothelial integrity. Early elevated plasma levels supply a valuable approach for prognostic parameters in patients. As STEC-HUS remains a rare disease predominantly affecting children, we encourage monitoring Angpt-2 levels for risk assessment.

In addition, we want to promote the concept of larger, prospective multicenter studies needed to confirm the reliability of prognostic values and possibilities of therapeutic intervention in the Angpt/Tie2 pathway.

## Supplementary Material

Supportive Figure 1: Western blot from HUVEC lysates stimulated with plasma of STEC-HUS patients (lo = low Angpt-1/-2 concentration, hi = high Angpt-1/-2 concentration). The sample with high Angpt-2 and low Angpt-1 (left column) shows a low pTie2 / tTie2 ratio. In contrast, the sample with low Angpt-2 and high Angpt-1 (right column) shows a higher pTie2 / tTie2 ratio.

## Figures and Tables

**Table 1:** Characteristics, chemistry values, and clinical symptoms in 48 patients with STEC-HUS mediated by *Escherichia coli* O104:H4.

Variable	Admission	Day 3
Demographics		
Number of patients (*n*, %)	48 (100)	
Age (years, median (IQR))	45 (27–57)	
Female sex (*n*, %)	35 (72.9)	
Positive test for Shiga toxin or EHEC (*n*, %)	45 (93.8)	
Onset of diarrhea (median (IQR))	2 (1–4)	
Medical history (*n*, %)		
Arterial hypertension	11 (22.9)	
Diabetes mellitus	2 (4.2)	
Coronary heart disease	2 (4.2)	
Chronic kidney disease	0 (0)	
Laboratory data (median (IQR))		
Creatinine (*μ*mol/L)	202 (120–335)	264.5 (184.8–344)
eGFR (mL/min/1.73 m^2^)	26 (15–51.5)	20 (13–33)
LDH (U/L)	1018 (749–1429)	886 (651–1142.3)
Platelets (/nL)	46 (32–64)	52 (23–72)
Hemoglobin (g/dL)	11.2 (9.8–12.4)	9.2 (8.3–10.4)
Angiopoietin-1 [ng/mL]	1.5 (0.7–2.1)	1.36 (0.95–2.29)
Angiopoietin-2 [ng/mL]	2.4 (2–3.2)	2.6 (1.8–3.28)
Clinical data (*n*, %)		
Complicated course	32 (66.7)	
Need of RRT	32 (66.7)	
Seizures	10 (20.8)	
Need of mechanical ventilation	9 (18.8)	
