# Supplementary material for: Involvement of Angiopoietin-2 and Tie2 Receptor Phosphorylation in STEC-HUS Mediated by Escherichia coli O104:H4
Source: Mediators Inflamm. 2015 Dec 24;2015:670248. doi: 10.1155/2015/670248 (PMC4706916; doi:10.1155/2015/670248)
Supplement: Supplementary file 1 — Supportive Figure 1: Western blot from HUVEC lysates stimulated with plasma of STEC-HUS patients (lo = low Angpt-1/-2 concentration, hi = high Angpt-1/-2 concentration). The sample with high Angpt-2 and low Angpt-1 (left column) shows a low pTie2 / tTie2 ratio. In contrast, the sample with low Angpt-2 and high Angpt-1 (right column) shows a higher pTie2 / tTie2 ratio. [file 670248.f1.pdf]

Figures

Supplementary Figure 1

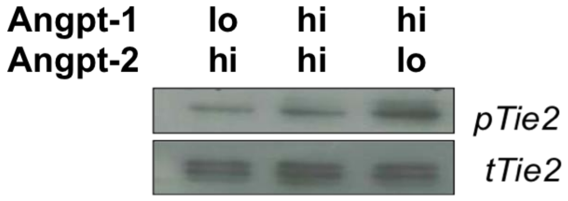

Figure Legends

Supplementary Figure 1:
Exemplary blot from HUVEC lysates stimulated plasma with from our patients (lo = low Angpt-1/-2 concentration, hi = high Angpt-1/-2 concentration). The patient with high Angpt-2 and low Angpt-1 (left lane) shows a weak pTie2 / tTie2 response in comparison with the patient with the more physiological balance, i.e. low Angpt-2 and high Angpt-1 (right lane).
